# Shonjibon cash and counselling: a community-based cluster randomised controlled trial to measure the effectiveness of unconditional cash transfers and mobile behaviour change communications to reduce child undernutrition in rural Bangladesh

**DOI:** 10.1186/s12889-020-09780-5

**Published:** 2020-11-25

**Authors:** Tanvir M. Huda, Ashraful Alam, Tazeen Tahsina, Mohammad Mehedi Hasan, Afrin Iqbal, Jasmin Khan, Gulshan Ara, Nazia Binte Ali, Saad Ullah Al Amin, Elizabeth K. Kirkwood, Tracey-Lea Laba, Nicholas Goodwin, Sumithra Muthayya, Munirul Islam, Kingsley Emwinyore Agho, John Hoddinott, Shams El Arifeen, Michael J. Dibley

**Affiliations:** 1 Sydney School of Public Health, The University of Sydney, Sydney, Australia; 2 International Centre for Diarrhoeal Disease Research, Bangladesh, Dhaka, Bangladesh; 3 University of Technology Sydney, Sydney, Australia; 4 The Sax Institute, Sydney, Australia, School of Public Health, The University of Sydney, Sydney, Australia; 5 Children’s Hospital Westmead Clinical School, Sydney Medical School, The University of Sydney, Sydney, Australia; 6 Western Sydney University, Sydney, Australia; 7 Cornell University, Ithaca, USA

## Abstract

**Background:**

Undernutrition is strongly associated with poverty - levels of undernutrition are higher in poor countries than in better-off countries. Social protection especially cash transfer is increasingly recognized as an important strategy to accelerate progress in improving maternal and child nutrition. A critical method to improve nutrition knowledge and influence feeding practices is through behaviour change communication intervention. The Shonjibon Cash and Counselling study aims to assess the effectiveness of unconditional cash transfers combined with a mobile application on nutrition counselling and direct counselling through mobile phone in reducing the prevalence of stunting in children at 18 months.

**Method:**

The study is a longitudinal cluster randomised controlled trial, with two parallel groups, and cluster assignment by groups of villages. The cohort of mother-child dyads will be followed-up over the intervention period of approximately 24 months, starting from recruitment to 18 months of the child’s age. The study will take place in north-central Bangladesh. The primary trial outcome will be the percentage of stunted children at 18 m as measured in follow up assessments starting from birth. The secondary trial outcomes will include differences between treatment arms in (1) Mean birthweight, percentage with low birthweight and small for gestational age (2) Mean child length-for age, weight for age and weight-for-length Z scores (3) Prevalence of child wasting (4) Percentage of women exclusively breastfeeding and mean duration of exclusive breastfeeding (5) Percentage of children consuming > 4 food groups (6) Mean child intake of energy, protein, carbohydrate, fat and micronutrients (7) Percentage of women at risk of inadequate nutrient intakes in all three trimesters (8) Maternal weight gain (9) Household food security (10) Number of events for child suffering from diarrhoea, acute respiratory illness and fever (11) Average costs of mobile phone BCC and cash transfer, and benefit-cost ratio for primary and secondary outcomes.

**Discussion:**

The proposed trial will provide high-level evidence of the efficacy and cost-effectiveness of mobile phone nutrition behavior change communication, combined with unconditional cash transfers in reducing child undernutrition in rural Bangladesh.

**Trial registration:**

The study has been registered in the Australian New Zealand Clinical Trials Registry (ACTRN12618001975280).

## Background

### Poverty and maternal and child nutrition

Undernutrition is strongly associated with poverty - levels of undernutrition are higher in poor countries than in better-off countries [1]. Of the world’s 736 million extreme poor (those living on less than US$1.90 a day or A$ 2.60) in 2015, 368 million, or half of the total, lived in India, Bangladesh, Nigeria, Democratic Republic of Congo and Ethiopia. All of these countries also recorded very high levels of undernutrition [2]. Besides, there is growing evidence that, within countries, the poor suffer from higher rates of undernutrition than the non-poor. In 2017 in Bangladesh, 24% of people lived below the poverty line compared to 49% in 2000 [3]. Reflecting this decline in poverty has been a corresponding improvement in child undernutrition. Despite this progress, there remains an estimated 21 million people living in extreme poverty, and child stunting remains high in rural populations (30.8% for under-5 children), and especially amongst the poorest 20% of households (40.2% for under-5 children) [4]. More than 20% of newborns in Bangladesh have low birth weight, which is amongst the highest levels worldwide [5, 6]. The COVID-19 pandemic is expected to increase undernutrition in vulnerable households due to an increase in food insecurity and reduced curative and preventive health services [7]. Adequate nutrition during the critical 1000-day window from conception to 2 years of age ensures long-term health and survival [8, 9]. Stunting of child linear growth is largely irreversible after the age of 2 years and leads to lower cognitive and educational attainment, and lower-income and socioeconomic status [8, 10, 11]. Improving maternal nutrition is critical to reducing low birth weight and improving child undernutrition.

### Cash transfers and maternal and child nutrition

The 2019 Lancet commission report, the 2008 and 2013 Lancet series on maternal and child nutrition, all strongly suggested the use of both nutrition-sensitive and nutrition-specific interventions to address the underlying determinants of poor nutrition [9, 12–14]. Social protection especially cash transfer (defined as the provision of assistance in the form of cash) is increasingly recognized as an important strategy to accelerate progress in improving maternal and child nutrition because this addresses structural factors such as poverty and social vulnerability. A growing body of evidence indicates that cash transfer can increase demand for preventive health care, food consumption and dietary diversity [15–19]. Cash transfer will be a key tool to mitigate the potential negative effects of COVID-19 on nutrition [7].

Cash transfer can either be conditional, where beneficiaries must comply with a set of conditions, or unconditional, where no conditions are required to receive payments. “Soft conditions” have been used where beneficiaries are not penalised for non-compliance with the conditions but instead are encouraged by community workers to comply. The premise underlying cash transfer programs for maternal and child nutrition is to increase food consumption to improve nutrition. Cash transfer programs have been shown to increase demand for preventive health care, food consumption, dietary diversity, and improve overall health outcomes, including child mortality [15–19]. Also, when targeted at women, cash transfer can promote women’s economic empowerment and enhance decision-making ability, with the overarching assumption that control over cash will lead to greater investment in children’s health and education [13, 20].

Currently, many countries in Latin America, Africa, and Asia are using cash transfer to reduce stunting, wasting and anaemia. There is some evidence of significant positive impacts on anthropometric measures such as child weight-for-age, height-for-age, and birthweight although it is not consistent across programs [9, 15, 21]. A meta-analysis to examine cash transfers on nutrition outcomes found small but non-significant impacts on height-for-age, and that conditional programs had similar results to unconditional cash transfers [13, 22]. As in most cases, the impact pathway is not analysed, so it is unclear why some cash transfer programs impact nutritional outcomes while others do not. There is also little evidence of the impact of cash transfer on important proximate and indirect outcomes such as caregiver feeding behaviours, practices and psychosocial care, the cost-effectiveness of cash transfer, the value of nutritional education, the value chains for nutrition, spill over effects (on women’s empowerment, local markets, food production, local governance, etc.) and sustained intergenerational impact. Compare to conditional cash transfer there has been less research on unconditional cash transfers. The theory underpinning this approach is that the poor are rational actors, and the provision of additional income will encourage uptake of desired behaviours through which they will eventually emerge out of poverty. Another major argument for the absence of conditions in the poor countries is the poorly developed supply side in health, which would not be able to cope with increased demand resulting from a CCT program. In addition, the capacity for implementing conditional transfer is also much weaker in most developing countries.

### Nutrition behaviour change communications

A critical method to improve nutrition knowledge and influence feeding practices is through behaviour change communication (BCC) intervention [23]. Recent studies of large-scale BCC interventions have found to improve IYCF practices in several countries [23, 24]. In Bangladesh, nutrition counselling is provided face to face by the frontline health workers. However, the coverage and the quality of such counselling remains an issue. In Bangladesh and other developing countries, mobile phones have been successfully used to inform, support and empower community health workers [25, 26]. Mobile phones are widely available in Bangladesh [26], and this channel has the potential to enhance communication about nutrition in large scale programs. There are currently more than ten recognised medical and health applications available in Bangladesh [27]. The Directorate General of Health Services of the government also has a health helpline 16,263 that provides advice as well as ambulance services [28]. In terms of mobile phone use, Bangladesh is the fifth largest market in Asia. Smartphone ownership and app use in Bangladesh is high, with 31% of the population possessing a smartphone, and this rate of use will likely increase to 75% by 2025. Adoption of mobile broadband is also expected to increase to 82% in 2025 [29]. These findings support that the population, including women of reproductive age, are increasingly turning to mobile health platforms to receive health information rather than relying on face-to-face and paper-based delivery methods. This trend towards the use of mobile health opens up an opportunity to reach women who are less likely to engage with health care providers or are yet to do so. Population-based telephone counselling combines ease of access and privacy with the advantages of in-person individual counselling. All of these reduce potential barriers to treatment seeking. Last but not least, mobile counselling may be standardised and cost-effective for the delivery of in-person counselling.

### Aims and hypothesis

The Shonjibon – Cash and Counselling (SCC) study aims to assess the effectiveness of unconditional cash transfers combined with a mobile application on nutrition counselling and direct counselling through mobile phone in reducing the prevalence of stunting (length-for-age < − 2 Z) in children at 18 months. The primary hypothesis is that in a community-based, cluster-randomised controlled trial of women from food insecure populations, mobile phone-based nutrition behaviour change communications (BCC) and unconditional cash transfers will reduce the prevalence of child stunting at 18 months by 6.2% (40.2% control – 34.0% intervention, or 16% relative reduction) compared to usual programs. The secondary hypotheses are that mobile phone nutrition BCC and unconditional cash transfers compared to the usual programs will: a) increase maternal caloric intake and dietary diversity; b) reduce the rate of low birth weight and small for gestational age newborns; c) improve breastfeeding and complementary feeding practices, including dietary diversity, d) improve women empowerment, and e) be cost-effective in reducing child undernutrition.

## Methods/design

### Study design

**Fig. 1:**
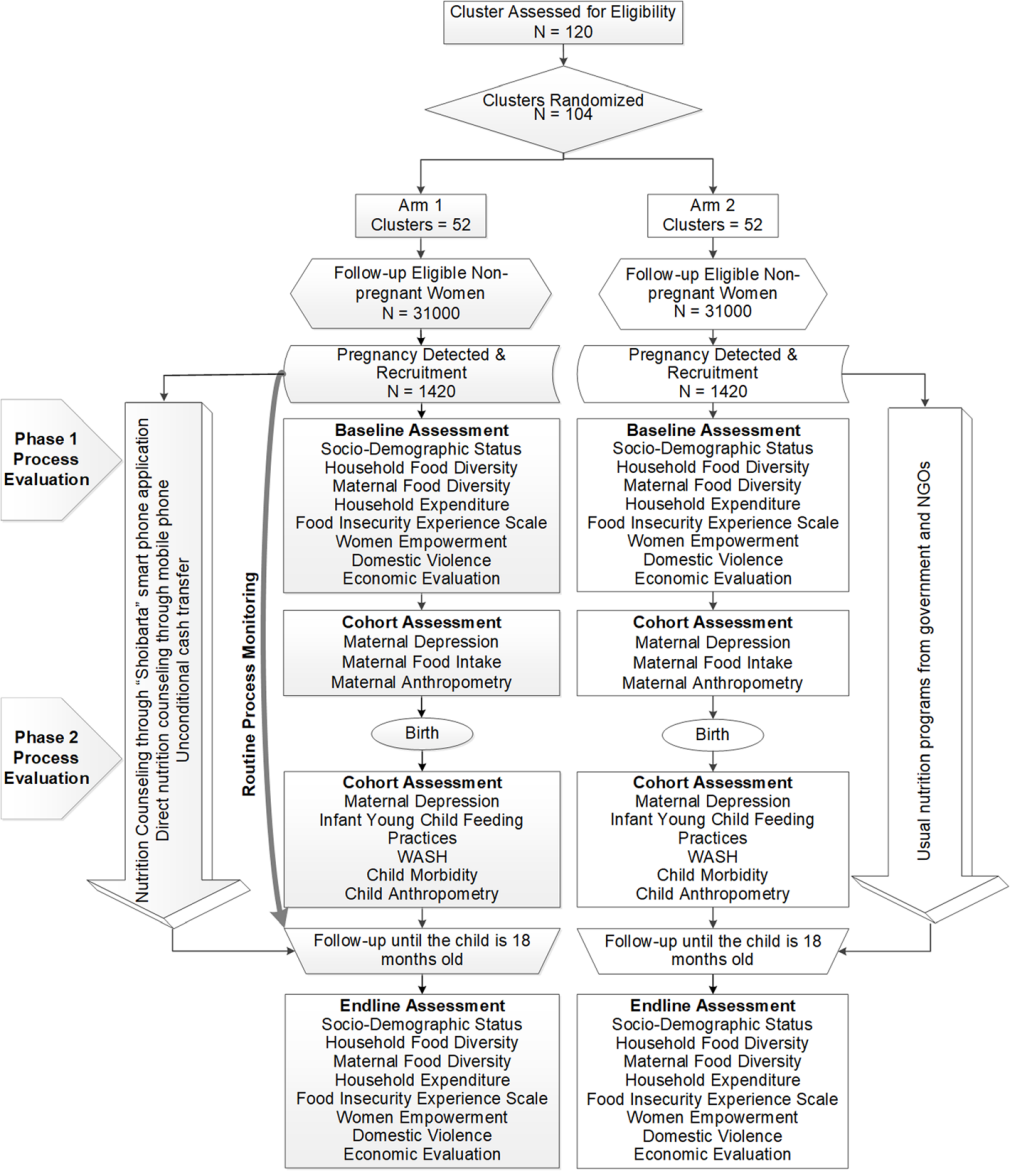
Study Flow

The study is a longitudinal cluster randomised controlled trial (cRCT), with two parallel groups, and cluster assignment by groups of villages. Children born to participants during the study will be the unit for analysis and clustering within villages will be taken into account. We have designed a mixed-methods process evaluation as part of the study to examine the factors and roles of the different processes that might influence the outcome. The cohort of mother-child dyads will be followed-up over the intervention period of approximately 24 months, starting from recruitment to 18 months of the child’s age (Fig. 1). This protocol has been written according to the recommendations of the SPIRIT 2013 statement [30]. Besides, the design of this clinical trial follows the requirements of the CONSORT statement.

### Study setting

**Fig. 2:**
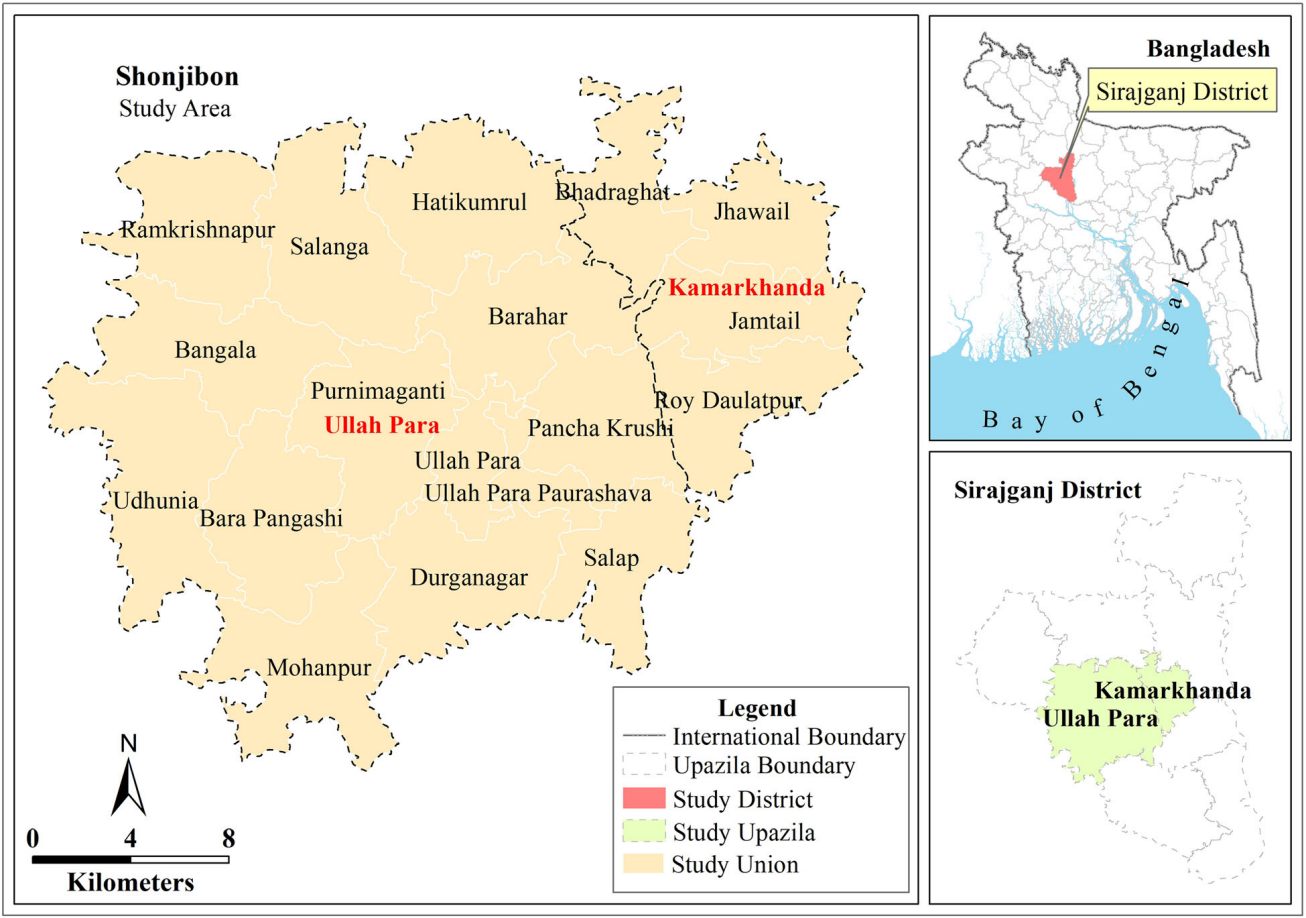
Shonjibon Cash & Counselling Study Area (Source: Author)

The study will take place in Ullahpara and Kamarkhanda subdistricts in the Sirajgonj district, which is in north-central Bangladesh (Fig. 2). It is a part of the Rajshahi Division. The Ullahpara and Kamarkhanda subdistricts have combined populations of 0.68 million [31]. According to the latest census, there are about 155,000 households in our study area; among them, 92% are in rural communities. The average size of a household in Sirajganj district is 4.3 persons. About 47% of households have electricity connection. In rural households, around 43% of households have electricity connection. The median age for the male is 21.9 years and female is 22.0 years. The literacy rate of the population 7 years and above is around 42%. The economy in the Sirajganj district is mostly agrarian and dependent on crop production. About 51% of the population relies on agriculture activities as their primary source of income. However, Sirajganj is also known for its textile industry. A large number of handloom and power loom industries have been established in the district over the last few years. About 40% of the households in the Sirajgonj are poor (i.e. households with total expenditure equal to or less than the amount required to buy food sufficient to provide 2122 Kcal per person per day and minimal non-food items) [32]. The prevalence of stunting in Sirajgonj is also high with > 40% of children 12–24 months stunted [4].

### Cluster recruitment

Each cluster consists of an average of 750 households. Of total 552 villages (451 in Ullahpara and 101 in Khamarkhanda) in our study area, we will include 389 accessible villages to give a total of 166 clusters, of which we will randomly select 104 clusters with an equal number of clusters in each arm of the trial (Fig. 1). The research team will request written approval for the study from the Directorate General of Health Service (DGHS) under the Ministry of Health & Family Welfare before allocating the clusters to the control or trial intervention.

### Eligibility criteria for inclusion of cluster

We excluded any potential villages if there are any other maternal and or neonatal interventions either by the government or non-government organisations in the area. We also excluded villages where access is challenging, for example, in flood-prone areas. All women aged between 15 to 49 years who are pregnant, whose gestational age is ≤90 days and who are permanent residents of the Shonjibon study area will be eligible.

### Participants recruitment

We will recruit married women aged between 15 and 49 years who have not had any permanent method of contraception (either the woman or her husband), are not using any family planning method and are a permanent resident of the study area.

### Formative research

We will conduct formative research to identify women’s and their families’ perceptions, attitudes and existing practices that are crucial to understanding to implement the intervention. The topics the formative research will include maternal and child nutrition and diet, use of mobile phones by women, spending money on food for mothers and children, women’s decision-making on family expenditure and child nutrition, and the role of persons within the family and the community. The findings will guide the production of the nutrition BCC material (text and video messages) to ensure they are culturally appropriate and acceptable to the community. We will test the messages and the mobile app in the community. The findings will help to finalise the baseline, follow up and adequacy survey tools.

We will conduct the research in three phases. *Phase one* involves in-depth interviews and free-listing exercises with women, their husbands and mothers-in-law to generate information about existing practices and perceptions that will help develop the BCC materials. *Phase two* includes group discussions and in-depth interviews with women to assess the clarity and understandability of the BCC materials. Once the draft messages and videos are developed and embedded in the Soi Barta app (the mobile app that will be used in the study and described below), we will conduct *Phase three* of the formative research. In this phase, field researchers will visit the women and run the app on the smartphone to assess the women’s response to the app and use of the smartphone. We will seek the women’s feedback and suggestions on the appropriateness, attractiveness, clarity and design issues in individual and group interviews. We will also explore women’s ideas on how to improve the app. The findings from these interviews will contribute to finalising the design of the app.

### Description of the intervention

#### Intervention arm

##### Nutrition behaviour change communication through a smartphone application

**Fig. 3:**
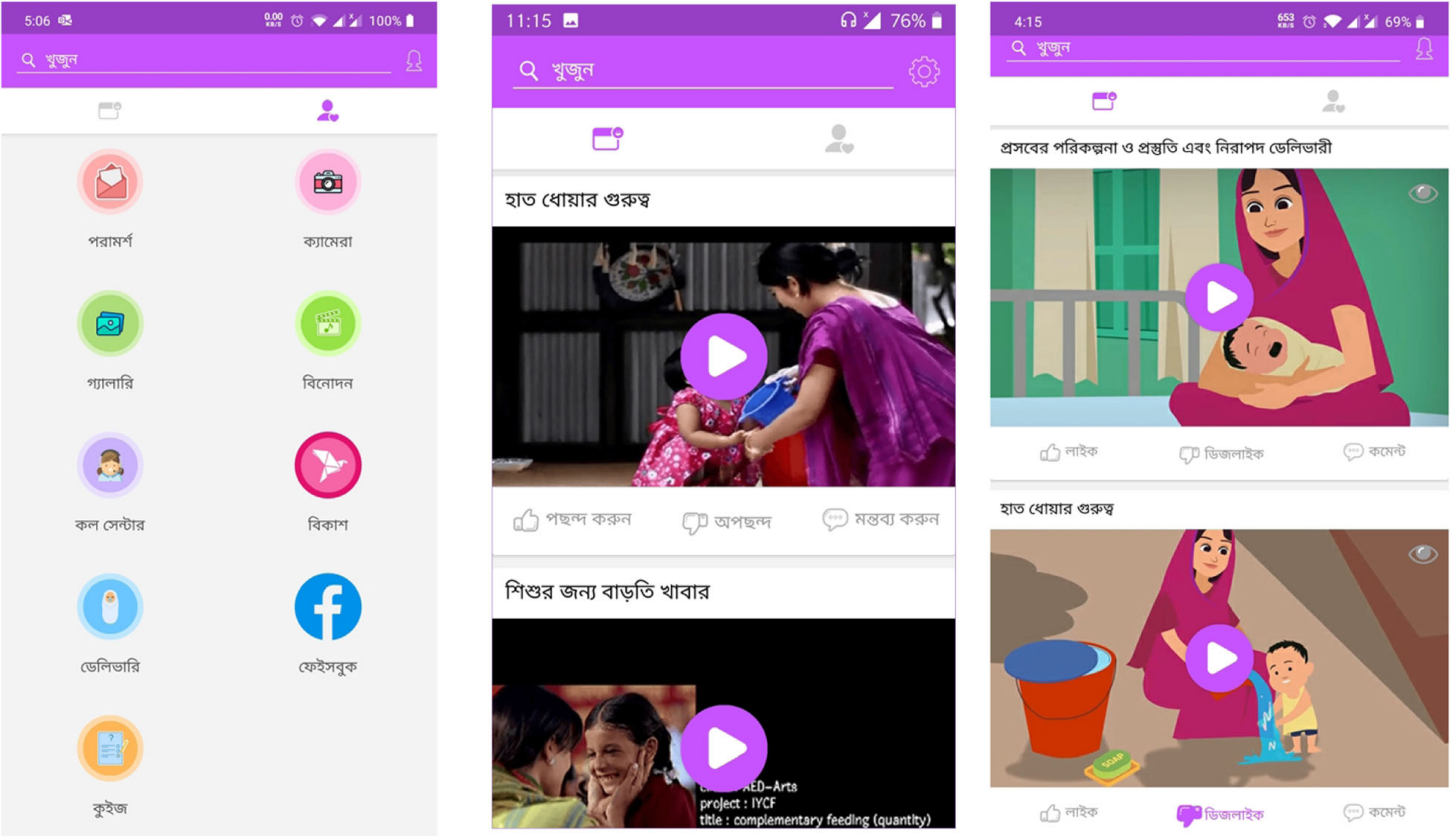
Soi Barta Mobile Application (Source: Author)

“Soi Barta” is a mHealth app designed to serve as a supportive health technology for mothers and young infants to meet their nutrition needs. The participants assigned to the intervention arm, with the help of the study team, will download the app on their mobiles and log in by inserting the registered mobile phone number. We will train the participants in the intervention arm to use the “Soi Barta” app during the enrolment visit. The app users will need to enter details of their last menstrual period or expected due date for the timing of notifications that are appropriate for the stage of their pregnancy. Users will receive two weekly animated/video/audio messages tailored to the stage of their pregnancy or the age of the child. The user receives the first section of the advice message as a push notification and is then encouraged to click on the notification to open the app and read the rest of the message (Fig. 3).

##### Nutrition behaviour change communication through mobile phone counselling service

We will establish a mobile phone counselling service (Soi Pushti Sheba) to provide nutrition counselling to the women in the intervention arm of the trial and other members of their households. The “Soi Pushti Sheba” team will comprise of experienced nutrition counsellors “Pushti Apa”. All nutrition counsellors managing the “Soi Pushti Sheba” will be trained to follow a contextual counselling and problem-solving process to listen to and understand our participants, and to focus on practical actions to address nutrition needs. The nutrition counsellors will actively listen to the mother’s belief and existing practices including susceptibility, severity, barriers to action and perceived benefit of action. The counsellors will use a nonjudgmental and empathetic disposition on mother’s perceptions. They will try to increase perceived susceptibility to and seriousness of a health condition by sharing information on the consequences of malnutrition on maternal and child health. They will also stimulate positive behaviour change by sharing knowledge on positive practices as a cue to action, highlighting motivational factors (sharing efficacy of positive nutrition practices in improving child growth and development) and enabling decision making through enhanced self-efficacy (empowerment through information sharing). The counsellors from “Soi Pushti Sheba” will use a life-course approach, from pre-conception and throughout the first 2 years of the child’s life. The counsellor will mainly focus on diet and micronutrient during pregnancy, on breastfeeding, and Infant and Young Child Feeding Practices (IYCF). The study will provide thirty-six 20-min fortnightly counselling sessions. The frequency and duration of the counselling sessions have been fixed based on our experience from a previous pilot study [33]. The core content for each counselling session will be synchronised with the “Soi Barta” mobile application to ensure we provide similar messages through both the channels at the same period of the life cycle.

##### Unconditional cash transfer

Through the participants’ mobile phone, we will deliver a cash transfer of US$12.5 per month, which is approximately 20% of the average monthly income of the poorest 40% of rural Bangladeshi households (~US$60/m). The well-developed food markets in rural Bangladesh will ensure food products are available for households to purchase. We will use a soft conditional approach where we will ask the mothers to listen to the messages and participate in counselling. However, we will make the cash transfers irrespective of the participant’s compliance. Project staff will follow up with mothers who do not participate to explore the reasons and reinforce the importance of the messages and counselling. Cash will be transferred directly to woman’s mobile bank accounts using the bKash mobile banking service.

#### Comparison arm

Routine nutrition counselling for pregnant women and mothers of under-five children by BRAC and government programs will continue with the same schedule in the control arm as in the intervention group at antenatal clinics, delivery, and immunisation clinics. We will provide mobile phones to the control arm to balance out any non-specific beneficial effects from the family having a mobile phone. We will contact the family by phone to minimise differential loss to follow up. However, experience with our previous trials indicates this is not likely to be a problem as low-income families’ value repeated examinations conducted as part of the trial evaluations.

### Trial outcomes

The primary trial outcome will be the percentage of stunted children (height-for-age < − 2 Z) at 18 m as measured in follow up assessments starting from birth. The secondary trial outcomes will include differences between treatment arms in (1) Mean birthweight, percentage with low birthweight and small for gestational age (2) Mean child length-for age, weight for age and weight-for-length Z scores from birth until 9 m, 12, 15 and 18 m (3) Prevalence of child wasting (weight-for-length < − 2Z) from birth until 18 m (4) Percentage of women exclusively breastfeeding and mean duration of exclusive breastfeeding monthly until 6 m (5) Percentage of children consuming > 4 food groups at 9, 12, 15 and 18 m (6) Mean child intake of energy, protein, carbohydrate, fat and micronutrients at 9, 12, 15 and 18 m (7) Percentage of women at risk of inadequate nutrient intakes in all three trimesters (8) Maternal weight gain per trimester (9) Household food security at baseline and end-line (10) Number of events for child suffering from diarrhoea, acute respiratory illness and fever (11) Average costs of mobile phone BCC and cash transfer, and benefit-cost ratio for primary and secondary outcomes.

### Outcome measurements

#### Anthropometry

To construct anthropometric indices and standard indicators including stunting (length-for-age < − 2 Z), wasting (weight-for-length < − 2 Z) and underweight (weight-for-age < − 2 Z) we will use the 2006 WHO Growth Standard [34]. We will also assess growth velocity using the same growth standard.

#### Infant feeding practices

To measure infant feeding practices, we will include questions about current breastfeeding status; ongoing use, timing and types of other liquids and solid foods; use of bottles for feeding; and information about who among family and friends are providing advice about infant feeding. We will assess breastfeeding self-efficacy with a validated five-level Likert scale [35]. To determine the level of social desirability and to adjust for this potential bias in our analyses, we will use the Marlowe-Crowne Social Desirability Scale [36].

#### Dietary intake

Dietary intake data will be collected three times during pregnancy; at the end of the first trimester, the end of the second trimester and 1 month after delivery. We will use a validated Food Frequency Questionnaire (FFQ) with three months’ recall to collect the usual dietary intake of pregnant women. The FFQ used in the MSAKI study in India will be adopted and validated in the local settings before use in the main trail. Portion sizes of foods will be quantified using standard local utensils. Recipes used to prepare meals will be recorded, including amounts of raw food used and the preparation methods. The dietary interviewers will use the 24-h recall method for collecting child dietary intake information by recording all foods and the quantity consumed by the child in the 24 h before the interview. Our analysis will compare the nutrient density of the infant diets with the desired levels, and the percentage of mothers at risk of inadequate nutrient intakes will be estimated using the fixed cut-point approach with WHO/FAO estimated average nutrient requirements.

#### Food security

We will assess household food security using the World Food Program’s Food Insecurity Experience Scale, an indicator of household food access, which combines data on dietary diversity and food frequency using 12 months recall [37].

#### Child morbidity

At scheduled monthly visits, trained interviewers will record maternal recall of symptoms in the preceding 2 weeks of common childhood illnesses (diarrhoea, acute respiratory illnesses and fever) using standard questions from Bangladesh Demographic and Health Survey (BDHS) [4].

#### Women’s empowerment

We will collect data on women empowerment at the baseline and end-line assessments of the trial using the Project-Level Women’s Empowerment in Agriculture Index (Pro-WEAI) questionnaire [38]. We will develop a composite indicator based on the following: 1) control over the use of income; 2) input in production decisions; 3) autonomy in income; 4) decision making power about women and children’s health and nutrition; 5) respect among household members and 6) attitudes toward IPV. The Pro-WEAI is used to measure empowerment of women, agency and inclusion in the agriculture sector [38], is a standardised, survey-based internationally validated index, and has been piloted in Bangladesh [38, 39].

#### Intimate partner violence

The Pro-WEAI includes questions about a woman’s attitude to intimate partner violence (IPV). Further data collection is required to determine precisely how cash transfers and nutrition behaviour change communication affects IPV. The additional questions ask about the personal experience rather than the women’s perceptions of IPV. We will take the questions for this assessment from the Violence Against Women and Girls Survey (Bangladesh 2015) [40].

#### Maternal depression

To measure any change in postpartum depression, we will use the Centre for Epidemiological Studies Depression Scale (CES-D). This twenty item self-report measure screens women for symptoms of emotional distress during pregnancy and the postnatal period.

#### Social, economic and demographic characteristics

Data for social, economic and demographic characteristics will be collected in baseline questionnaires using Demographic and Health Survey methods which include an inventory of household assets to construct a wealth index [41] and the mother’s socio-demographic and reproductive characteristics.

#### Economic evaluation

We will conduct an economic evaluation, from the societal perspective, to assess the costs and cost-effectiveness of the intervention compared to usual care. Data sources and collection methods for the costs will include project records and surveys conducted throughout the follow-up period, and we will follow the WHO Costit modules to estimate the *costs of delivering the intervention* (start-up costs, staff, training, mobile phone service, cash transfers). We will estimate the average costs per individual in each treatment group throughout the follow-up. The economic evaluation will consist of a cost consequence analysis (CCA) and cost-effectiveness analysis (CEA). Cost consequences will include all outcomes listed in the study objectives. The CEA will focus on the incremental costs per Disability-Adjusted Life Years (DALYs) gained in terms of reductions in stunting.

#### Health-related quality of life (HRQoL)

We will use the WHO-BREF tool at the baseline and end-line data collection. The tool has 26 questions from the WHO-100 QoL questionnaire covering various dimensions, each of which has five levels with categories depending on the type of questions asked.

### Process evaluation

We will conduct a mixed-methods ongoing process evaluation to examine the implementation of the interventions, including delivery of messages, cash and nutrition counselling, to identify the contextual factors that affect the interventions, and to assess the reach of the interventions [42]. We will develop a Program Impact Pathway (PIP) to illustrate the possible routes of achieving the impact of the different intervention elements. The PIP will identify the possible causal links between the interventions and their intended impacts. It includes defining changes, linking processes, and identifying indicators to monitor progress towards the expected impacts. Thus, the PIP will guide the process evaluation data collection. The data will be collected through in-depth interviews and focus group discussions with intervention recipients (mothers of infants, their husbands) and implementers (project officers, managers, and counsellors). We will collect the data longitudinally in three phases. The first phase of data collection will take place within 1 month of the onset of the intervention, while the second and the second and third phases of data collection will be at midline and end-line of the intervention. These methods will generate information about beneficiaries’ response to the intervention, barriers to comply with the nutrition messages and use of cash, and any contextual factors that may influence the outcome. We will also collect information on variations in the accessibility and response to the intervention among the participating households or clusters and underlying factors for the differences will also be collected. In addition to the qualitative data, we will collect quantitative information as part of the regular project monitoring to assess intervention adequacy and reach. We will use the process evaluation data to make necessary adjustments to the intervention and to keep the program ‘on-track’ (formative use) and to interpret and explain outcome results (summative use) at the end of the trial [43].

### Sampling frame and sample size

We require a sample size of 2184 mother-infant pairs (1092 per group) from 104 clusters recruited over 6 months to demonstrate a 6.2% difference in stunting prevalence between treatment groups (40.2% in control to 34.0% in the intervention or 16% relative reduction). The assumed stunting reduction is similar to that observed in a recent study in Bangladesh for face-to-face nutrition BCC plus cash transfers [unpublished data]. Taking account of the potential loses noted below; we would need to recruit 2840 pregnant women to ensure this sample size for the outcome assessment. In this calculation, we used 80% power, a standard two-tailed 5% significance level, a design effect of 1.28 (based on an estimate for stunted children age 12–24 months from the poorest 40% of households in the 2014 BDHS data and an intracluster correlation coefficient (ICC) of 0.0051 assuming a cluster size of 20. This sample size would detect a five-percentage point difference in the prevalence of low birth weight (LBW) between treatment groups with 80% power assuming an ICC of 0.01 and prevalence of LBW of 25% in the control group.

To calculate the estimated sample size, we made several assumptions. We expect the prevalence of stunting (length-for-age < − 2 Z) of 40.2% in control clusters according to latest round of Bangladesh Demographic and Health Survey [4]. Each cluster will have two villages with an average population of 3000. Considering the crude birth rate of 23/1000 population over 12 months [44], we expect that the average number of births will be approximately 34 over 6 months in each cluster. We expect to reach 90% of births though our surveillance system, which means that approximately 31 mother-infant dyads would be available per cluster over 6 months. From our experience in earlier trials, we expect to lose about 10% of pregnancies before birth, 5% of the recruited women will migrate out from the study area and 10% of pregnant women will deliver outside their village. We expect further loses from child deaths in the first 2 years of life.

### Recruitment and consent of women

We will conduct a census in all households in the study area and record the names and contact details of all consenting women in an electronic system with a unique I.D. number. Our surveillance worker will then conduct door-to-door bi-monthly visits to identify women missing two menstrual periods in a row. All such woman will undergo a pregnancy test with a sensitive pregnancy urine test kit (Excel®). We will invite women to participate who test positive in the study with informed written consent. Based on experience with our previous research with similar design and settings, we expect at least 95% of mothers will consent.

### Randomisation and allocation of treatments

The treatments will be assigned to eligible clusters using a fixed randomisation scheme with a uniform allocation ratio of treatments, stratified by unions. We will use Stata® software to generate the random allocation sequence. Stratification of clusters based on unions will ensure geographic balance across the study area neutralising the effect of contextual factors. It will also mean that both the intervention and control groups will receive similar maternal and child health and nutrition services from the government. The only difference between the treatment arms will be the mobile phone nutrition BCC with cash transfers in the intervention arm. This design will control for potential confounding factors (observed and unobserved) as we will randomly allocate an adequate number of clusters (104 in total) to one of the groups. Contamination of interventions will be constrained by geographic separation of clusters, and by the nature of the interventions (mobile phones and cash). The nature of the intervention precludes the masking of the treatments.

### Evaluation

#### Data collection team

To minimise assessment bias because of a lack of trial blinding, we will separate the intervention and evaluation staff. During the training of the evaluation team, we will not reveal the trial hypotheses or interventions. Each surveillance worker will record the details of married women aged 15–49 years in her village who consent to menstrual monitoring and monthly follow up. She will inform the study health worker if a woman reports two missed periods. The study health worker will then visit, validate the pregnancy through a pregnancy kit and enrol the woman. For surveillance and quantitative data collection, there will be 30 surveillance workers and 15 data collectors who will be supervised by three field supervisors. Also, we will form seven teams with each team consisting of two persons for child anthropometry measurements. There will also be two senior-level field data collectors for the 24-h dietary intake measurements in a sub-sample. At the central level, two senior researchers and three junior researchers will provide overall supervision and management of field activities and data collection.

#### Data collection schedule

**Fig. 4:**
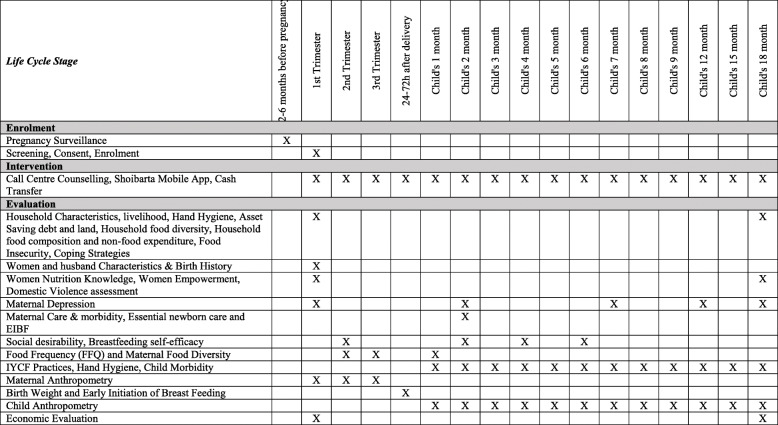
Schedule of enrolment, intervention and assessment

Standard questions on infant feeding practices will be collected monthly until 9 months and every 3 months after that until 18 months (Fig. 4). Along with infant feeding practices, we will also record maternal recall of symptoms from the preceding 2 weeks of common childhood illnesses (diarrhoea, acute respiratory illnesses and fever) using standard questions from BDHS [4, 44]. Further, at baseline, we will collect data on social, economic and demographic characteristics along with household food security and maternal nutrition knowledge. We will measure household food security and maternal nutrition knowledge at end-line. Specially trained interviewers will collect 24-h dietary recalls using standard methods from the mothers at baseline, in the third trimester and for the children at 9, 12, & 18 m (Fig. 4). Anthropometry will be collected within 72 h of birth and monthly until six months and then at 9, 12, & 18 m (Fig. 4). Trained research assistants will collect weight and height measurements using established methods [45]. We will conduct these standardisation exercises before and during data collection.

#### Electronic data collection tool

The team in the field will use an Android Tablet interactive data collection tool for data capture. The software navigates the data collectors during each specific evaluation interview. Once they complete an interview, the data are uploaded to the server at the International Center for Diarrheal Disease Research, Bangladesh (icddrb) and merged with existing data. A copy of the data remains on the tablet. Tablets are synchronised with the server each day. We will monitor data regularly, to allow real-time adjustments for any data inconsistencies. A team of four, led by a lead researcher, will collect qualitative data. They will record the interviews then transcribe and translate them into English within a week of data collection.

### Statistical analyses

Data analyses will be by intention to treat and by a statistician blinded to the treatment group to reduce interpretation bias. We will conduct the analyses at the mother-infant dyad level and will adjust for cluster randomisation [46]. In primary analyses, we will compare the prevalence of stunting (length-for-age < − 2 Z) in children at 18 months using Pearson’s chi-square tests and 95% confidence intervals for the group difference adjusted for clustering. In secondary analyses, we will examine each outcome variable (birth weight, feeding patterns, and mean nutrient intakes) taking account of the repeated measurements within children by using separate mixed models. We will use linear mixed models for continuous outcomes (e.g. height-for-age Z) and generalised linear mixed models for non-continuous outcomes (e.g. binary outcomes such as exclusive breastfeeding). Models will include treatment group as a fixed effect, infants as a random effect to account for repeated measurements, and community-cluster as a random effect to account for cluster effects. We will use STATA® for all analyses.

### Economic analyses

An economic evaluation will estimate the incremental cost-effectiveness ratio (ICER) from a societal perspective for mobile phone nutrition BCC plus cash transfer intervention compared to usual care. We will conduct the economic assessment in two phases: 1) Trial based evaluation to assess incremental costs and effects of adding this intervention to usual care; 2) Modelled analysis for estimating lifetime costs & disability for mothers and children. Costing templates (Costit) will be followed to record the financial and economic costs of our intervention. We will include all items of the program and household level costs in the tool. For the program cost, we will track all expenses incurred throughout the implementation period. We will distinguish fixed costs and variable costs. The fixed costs will cover the costs of developing the BCC content and mobile application, the costs associated with the cash transfer, the cost of the mobile phone. Variable costs will include the costs for intervention (reaching out to women), the maintenance of mobile phones, monitoring and troubleshooting. At end-line, we will use the program summary costs, which includes costs incurred for program implementation and for care-seeking at an individual level, to calculate the total cost. We will collect the costs of care-seeking during baseline, end-line and follow up visits during the intervention period. We will disaggregate costs of illness into two components – direct costs and indirect costs. Direct costs will include household expenditures related to treatment-seeking (medical expenses, such as physician’s fee, drug costs, cost of hospital stay - both for the patient and accompanied person) along with non-medical expenses such as transport costs. Indirect costs will include information on loss of household productive labour time (monetised loss of working hours) for patients and accompanying household members.

### Data monitoring

#### Electronic data monitoring tool

We will develop an online monitoring system, which will provide real-time information on enrolment status, adherence of the data collectors with the evaluation schedule and quality of data for critical variables. This system will allow the field supervisors to monitor the performance of individual data collectors. All reports will be auto generated from the system and will be available for investigators’ assessments.

#### Field monitoring and standardisation

In the field, our Field Research Assistant will observe the interviews and will take duplicate measurements to calculate intra- and inter-observer Technical Error of Measurement (TEM). We will also conduct standardisation sessions for field staff. In each session, we will ask data collectors to take duplicate measurements (weight, height, MUAC for women; weight, length, head circumference for children) on ten volunteers. We will calculate the intra- and inter-observer Technical Error of Measurement (TEM) and use the results to identify poor data collectors for re-training and additional field support.

#### Data safety and monitoring board

We will form an independent data safety and monitoring board (DSMB). It will assess the interim data, data quality, data completeness, adequacy of compliance with goals for recruitment and retention, and factors that might affect the study outcome or compromise the confidentiality of the trial data. We will notify the DSMB of any unintended effects of the trial intervention.

#### Access to data

All data will be accessible to the study investigators. The investigators will have the right to analyse and publish data. We will only share datasets externally after we have removed all personally identifiable information and with every reasonable effort to keep the identification of study subjects in the strictest confidence.

#### Dissemination plan

The research team will share the lessons learned from the study widely throughout Bangladesh and among global audiences. The authors will organise dissemination seminars to share the findings with the relevant stakeholders. We will present the intermediate and immediate results which support the hypotheses generated from the trial at national and international conferences and publish them in conference proceedings. We will publish the analysis of the outcomes in the form of internal documents, working papers, and in international peer-reviewed journals. We will base authorship eligibility on recommendations of the international committee for medical journals editors (ICMJE).

## Discussion

This study protocol describes a cluster randomised controlled trial of a cash and nutrition education-based intervention, to improve the nutritional status of children less than 2 years of age to reduce stunting. Poor nutritional status of children is a major global public health problem in low- and middle-income countries [47] accounting for 35% of under-5 child deaths and 33% of the disease burden in this age group [47]. It is a significant problem in Bangladesh, which has > 20% low birth weight [5], the second-highest prevalence of child underweight globally [48] and a very high prevalence of stunting [4]. Despite falls in poverty, the levels of extreme poverty, food insecurity and vulnerability remain high with 21 million people living in extreme poverty [3]. Developing effective combinations of nutrition-specific and nutrition-sensitive interventions will be integral components of efforts to develop sustainable development policies and programs for nutrition and to prevent stunting [13]. In recent years, there has been an increasing interest in the use of cash transfer to reduce malnutrition. In a previous study, we found that cash alone is unable to prevent child stunting, and only the combination of cash transfers plus nutrition BCC improved child growth to reduce stunting. However, both cash transfer and face-to-face nutrition counselling are expensive. So, it is of high priority to find a cost-effective delivery platform and to produce strong evidence that the interventions will work best in the Bangladesh country context. Without strong evidence, it would be difficult for policymakers to scale up such an intervention nationwide. The proposed trial will provide high-level evidence of the efficacy and cost-effectiveness of mobile phone nutrition behavior change communication, combined with unconditional cash transfers in reducing child undernutrition in rural Bangladesh. This trial of an innovative approach to enhancing the impact of cash transfers on child nutrition will be a leading study to show an efficacious and cost-effective approach to reducing maternal and child undernutrition in a low-income food insecure population.

## Data Availability

Not applicable.

## Abbreviations

ERC: Ethical Review Committee
HREC: Human Research Ethics Committee
NHMRC: National Health and Medical Research Council
